# Use of Generative AI for Mental Health Advice Among US Adolescents and Young Adults

**DOI:** 10.1001/jamanetworkopen.2025.42281

**Published:** 2025-11-07

**Authors:** Ryan K. McBain, Robert Bozick, Melissa Diliberti, Li Ang Zhang, Fang Zhang, Alyssa Burnett, Aaron Kofner, Benjamin Rader, Joshua Breslau, Bradley D. Stein, Ateev Mehrotra, Lori Uscher Pines, Jonathan Cantor, Hao Yu

**Affiliations:** 1 RAND, Arlington, Virginia; 2 Mass General Brigham, Boston, Massachusetts; 3 Harvard Medical School, Boston, Massachusetts; 4 RAND, Santa Monica, California; 5 RAND, Pittsburgh, Pennsylvania; 6 Department of Population Medicine, Harvard Pilgrim Health Care Institute, Boston, Massachusetts; 7 Boston Children’s Hospital, Boston, Massachusetts; 8 Brown University School of Public Health, Providence, Rhode Island

## Abstract

This cross-sectional study surveyed US adolescents and young adults on their use of generative artificial intelligence (AI) for mental health advice, including frequency and perceived helpfulness.

## Introduction

Since the launch of large language model (LLM) chatbots, use of this form of generative artificial intelligence (AI) has grown rapidly, especially among adolescents and young adults.^1^ Concurrently, the US is experiencing a youth mental health crisis.^2^ In the past year, 18% of adolescents aged 12 to 17 years had a major depressive episode; 40% of these received no mental health care.^3^

It is unclear how many adolescents and young adults use LLM chatbots for advice or help when experiencing emotional distress. We report results from the first nationally representative survey of US adolescents and young adults aged 12 to 21 years examining the prevalence, frequency, and perceived helpfulness of advice from generative AI when feeling sad, angry, or nervous.

## Methods

This cross-sectional study was approved by Harvard’s institutional review board and follows the STROBE reporting guideline. Informed consent was collected. Between February and March 2025, we surveyed youths from RAND’s American Life Panel and Ipsos’ KnowledgePanel, which use random sampling from population frames of US households. Produced survey statistics generalize to the population of US English-speaking youths aged 12 to 21 years with internet access. The survey assessed whether respondents had ever used generative AI (yes or no); whether they had sought *advice or help* from generative AI when feeling sad, angry, or nervous (yes or no; hereafter, *mental health advice*); frequency of seeking such advice; and perceived helpfulness of such advice.

To ensure comprehension among youths as young as 12 years, the focal question (No. 2) used plainspoken terms describing common emotional states associated with mental health needs (feeling sad, angry, or nervous). We also explicitly asked about generative AI use “for advice or help” in those circumstances. Respondents were given a definition of generative AI and examples (ChatGPT [OpenAI], Gemini [Google AI and DeepMind], and My AI [Snap]). Details on panel construction, survey questions, and methodology are in eMethods 1-3 in Supplement 1.

We calculated survey-weighted percentages and conducted multivariable logistic regression to examine factors associated with generative AI use for mental health advice: respondent age, sex, and race and ethnicity; highest parental education level; parental marital status; and census region. Analyses used Stata version 19.5 (StataCorp).

## Results

Of 2125 individuals contacted, 1058 responded (response rate, 49.8%; 37.0% aged 18 to 21 years; 50.3% female; 13.0% Black, 25.2% Hispanic, and 51.3% White) (Table). In total, 13.1% of respondents reported using generative AI for mental health advice (Figure), with higher rates among those aged 18 to 21 years (22.2%). Among users, 65.5% sought advice monthly or more often and 92.7% found the advice somewhat or very helpful. All results percentages are weighted.

**Table.** Descriptive Characteristics of Survey Sample

Sample characteristic	Participants, No. (%)^a^	
	Sample size	Weighted sample size^b^
Sex		
Male	493 (46.6)	20 456 992 (49.7)
Female	565 (53.4)	20 687 859 (50.3)
Age, y		
12-14	434 (41.0)	12 338 928 (30.0)
15-17	477 (45.1)	13 594 940 (33.0)
18-21	147 (13.9)	15 210 983 (37.0)
Race and ethnicity^c^		
Black	103 (9.7)	5 327 470 (13.0)
Hispanic	258 (24.4)	10 372 657 (25.2)
White non-Hispanic	576 (54.4)	21 118 502 (51.3)
Other	121 (11.4)	4 326 222 (10.5)
Parent education		
≤High school degree	304 (28.7)	13 970 346 (34.0)
Some college	314 (29.7)	13 750 439 (33.4)
≥Bachelor’s degree	440 (41.6)	13 424 066 (32.6)
Parent current living status		
Married	702 (66.4)	21 646 464 (52.6)
Separated, divorced, or widowed	127 (12.0)	4 129 086 (10.0)
Never married	229 (21.6)	15 369 300 (37.4)
Census region		
Northeast	203 (19.2)	6 933 850 (16.9)
Midwest	197 (18.6)	8 440 499 (20.5)
South	443 (41.9)	16 333 616 (39.7)
West	215 (20.3)	9 436 885 (22.9)

^a^ Counts of adolescents and young adults who responded to generative artificial intelligence survey questions.

^b^ All percentages and regression results use survey weights.

^c^ Race and ethnicity were self-reported. Respondents were asked 2 questions: Do you consider yourself Hispanic or Latino? Do you consider yourself primarily American Indian or Alaska Native, Asian or Pacific Islander, Black or African American, or White? White indicates they responded no to the first and White to the second question. Black indicates they responded no to the first and Black to the second question. Other indicates they responded no to the first and American Indian or Alaska Native or Asian or Other Pacific Islander to the second question.

**Figure.**
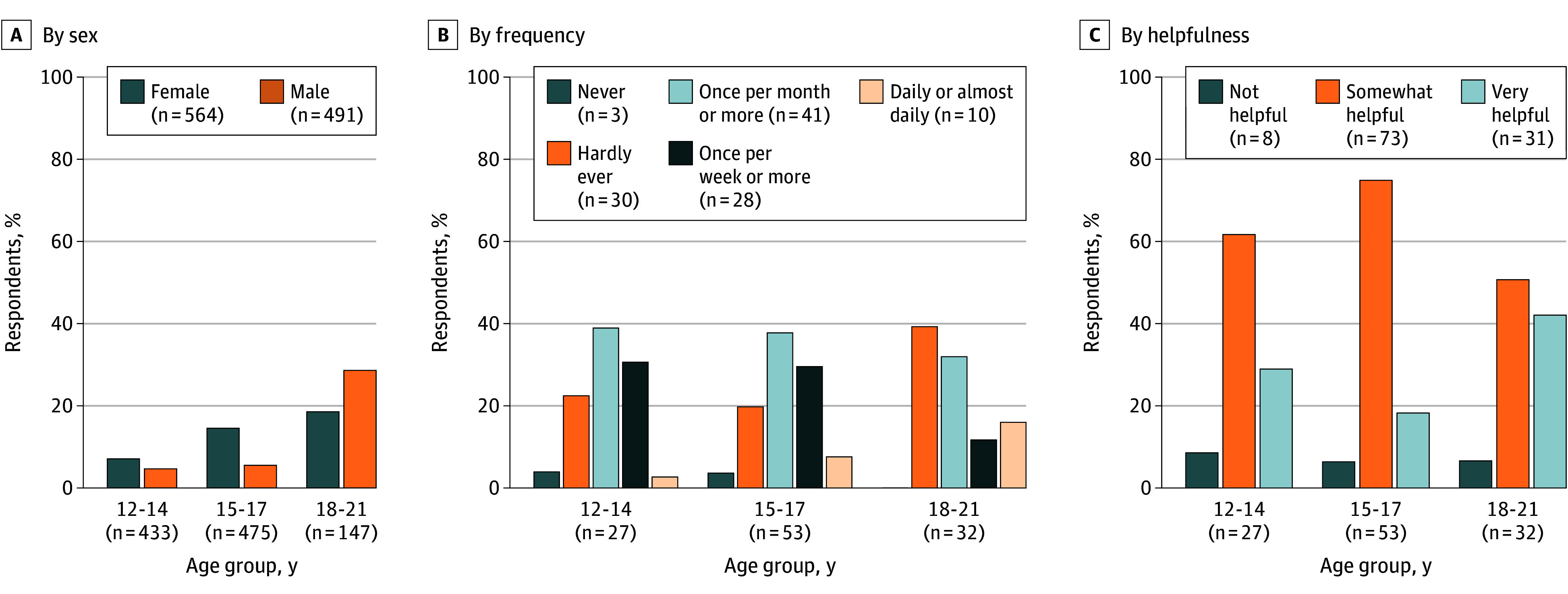
Use of Generative Artificial Intelligence for Mental Health Advice Weighted percentages represent respondents reporting in the affirmative to the response category. The number of observations is unweighted. Mental health advice was defined in the survey as “advice or help when feeling sad, angry, or nervous.”

In multivariable regression analyses, generative AI use for mental health advice was higher among youths ages 18 to 21 years (adjusted odds ratio [aOR], 3.99; 95% CI, 1.90-8.34; *P* < .001) compared with younger adolescents. Black respondents were less likely to report the advice as helpful (aOR, 0.15; 95% CI, 0.04-0.65; *P* = .01) compared with White non-Hispanic respondents. No other differences were statistically significant.

## Discussion

In this cross-sectional study’s nationally representative survey, we found that 13.1% of US youths, representing approximately 5.4 million individuals, used generative AI for mental health advice, with higher rates (22.2%) among those 18 years and older. Of these 5.4 million users, 65.5% engaged at least monthly and 92.7% found the advice helpful.

High use rates likely reflect the low cost, immediacy, and perceived privacy of AI-based advice, particularly for youths unlikely to receive traditional counseling.^4^ However, engagement with generative AI raises concerns,^5^ especially for users with intensive clinical needs, given difficulties in establishing and using standardized benchmarks for evaluating AI-generated mental health advice and limited transparency about the datasets training these models.^6^ Furthermore, Black respondents reported lower perceived helpfulness, signaling potential cultural competency gaps.

Study limitations include modest sample size, potential nonresponse and survey response biases, and an absence of information on the generative AI used and types of advice sought. Our sample size was particularly small (147 respondents) for ages 18 to 21 years, and findings should be interpreted with caution. Our findings are generalizable only to English speakers aged 12 to 21 years with internet access. Additionally, the survey did not include measures of diagnosed mental illness. Future research should examine use rates among children and youths with mental health conditions and associations with mental health outcomes.

## Notes

Supplement 1. **eMethods 1.** Survey Methodology **eMethods 2.** Statistical Approach **eMethods 3.** List of Questions

Supplement 2.
Data Sharing Statement
