# Supplementary material for: Use of Generative AI for Mental Health Advice Among US Adolescents and Young Adults
Source: JAMA Netw Open. 2025 Nov 7;8(11):e2542281. doi: 10.1001/jamanetworkopen.2025.42281 (PMC12595529; doi:10.1001/jamanetworkopen.2025.42281)
Supplement: Supplement 1. — eMethods 1. Survey Methodology eMethods 2. Statistical Approach eMethods 3. List of Questions [file jamanetwopen-e2542281-s001.pdf]

Supplemental Online Content

McBain RK, Bozick R, Diliberti M, et al. Use of generative AI for mental health advice among US adolescents and young adults. *JAMA Netw Open*. 2025;8(11):e2542281. doi:10.1001/jamanetworkopen.2025.42281

eMethods 1. Survey Methodology

eMethods 2. Statistical Approach

eMethods 3. List of Questions

This supplemental material has been provided by the authors to give readers additional information about their work.

© 2025 McBain RK et al. *JAMA Network Open*.

eMethod1. Survey Methodology

Procedures

Participants were drawn from members of RAND's American Life Panel (RAND-ALP) and Ipsos' KnowledgePanel® (Ipsos-KP), both of which are nationally representative survey panels of adults in the United States. [19] Unlike clinical-based samples, convenience samples, or samples of medical records, these panels were constructed using probability-based sampling methods applied to the universe of housing units in the U.S. Therefore, data from these panels can support inference to the online population of English-speaking U.S. youth between the ages of 12 and 21. RAND-ALP and Ipsos-KP panelists regularly complete online surveys on an array of topics including education, employment, and health. Surveys are administered online, in English, and participants receive financial compensation. Adults aged 18 and older are initially enrolled in the RAND-ALP and the Ipsos-KP based on random samples of household unit listings from the U.S. Postal Service's Delivery Sequence File. Adult panel members consent to the participation of eligible minors within their household for surveys focused on youth. For this study, 2,125 youth were invited to participate, following parental consent and adolescent assent for respondents aged 17 or younger. The survey was administered to both panels in February and March 2025. 49.8% of panelists invited to take the survey completed it, which is in line with other major population-based health surveys. For context, the U.S. Centers for Disease Control and Prevention administers two national surveys to random samples of U.S. households: The National Health Interview Survey (NHIS) and the National Health and Nutrition Examination Survey (NHANES). These two surveys, which collect health condition and health behavior data, are used to produce official prevalence rates for the country. In the 2024 NHIS, response rates for adults and children were 48% and 46%, respectively. In the 2021-23 NHANES, response rates for 12-15 year-olds and 16-20 year-olds were 37% and 32%, respectively. We applied survey weights to our analytic sample to align with U.S. population distribution benchmarks of age, race/ethnicity, sex, and geographic region from the 2023 American Community Survey.

Measures

We developed three survey items that examined large language model (LLM) use for mental health advice. Respondents were first informed that chatbots or apps that answer questions are example uses of generative AI, and that examples include ChatGPT, Google Gemini, and SnapChat My AI. Respondents were asked whether they use LLMs. Among those who use LLMs, respondents reported whether they had ever used generative AI for advice or help when feeling sad, angry, or nervous (yes/no). In our analyses, we categorized those who never used LLMs as also never having used generative AI for mental health advice. Respondents who reported ever using generative AI for mental health advice then indicated their frequency of such use (never, hardly ever, at least once a month, at least once a week, daily/almost daily) and the perceived helpfulness (very helpful, somewhat helpful, or not helpful).

Individual demographic variables collected from members of both panels included sex, age, and race/ethnicity. Additionally, household characteristics such as parent's highest level of education and parental marital status (never married, married, currently not married) are included. Lastly, we linked geographic identifiers for each respondent to their U.S. census region.

© 2025 McBain RK et al. *JAMA Network Open*.

eMethod 2. Statistical Analysis

We estimated three separate multivariable regression models. Survey weights were used for all regressions. First, we performed a multivariable logistic regression to predict whether respondents used generative AI for advice or help when feeling sad, angry, or nervous. The sample included all survey respondents. The explanatory variables (fixed effects) were: the respondent's sex, age, and race/ethnicity; the highest level of education attained by the respondent's parent; the parent's current marital status; and the census region.

Second, we performed an ordinal logistic regression to predict how frequently respondents used generative AI for advice when feeling sad, angry, or nervous. The sample was restricted to survey respondents who stated that they used generative AI for advice or help when feeling sad, angry, or nervous. The outcome variable had three levels: (1) "never," or "hardly ever," (2) "at least once a month," (3) or "at least once a week," or "daily, or almost daily." The same set of explanatory variables was incorporated into the model.

Finally, we performed another ordinal logistic regression to predict how helpful respondents found generative AI's advice when feeling sad, angry, or nervous. The sample was restricted to survey respondents who stated that they used generative AI for advice or help when feeling sad, angry, or nervous. The outcome variable ranged from "not helpful," to "somewhat helpful," to "very helpful." Again, we incorporated the same explanatory variables as in the previous models.

© 2025 McBain RK et al. *JAMA Network Open*.

eMethod3. List of Questions

Chatbots or apps that answer your questions are called **generative AI**. Examples of generative AI include ChatGPT, Google Gemini, and Snap's My AI.

(1) Do you use generative AI?

a. Yes (go to question 2)
b. No (skip to questions in next section of survey)

(2) Do you ever use generative AI for advice or help when you're feeling sad, angry, or nervous?

a. Yes (go to questions 3 and 4).
b. No (skip to questions in next section of survey)

(3) How often do you use generative AI for advice or help when you're feeling sad, angry, or nervous?

a. Never
b. Hardly ever
c. At least once a month
d. At least once a week
e. Daily or almost daily

(4) How helpful is generative AI when you feel sad, angry, or nervous?

a. Very helpful
b. Somewhat helpful
c. Not helpful

© 2025 McBain RK et al. *JAMA Network Open*.
