# Supplementary material for: Use of Generative AI for Mental Health Advice Among US Adolescents and Young Adults
Source: JAMA Netw Open. 2025 Nov 7;8(11):e2542281. doi: 10.1001/jamanetworkopen.2025.42281 (PMC12595529; doi:10.1001/jamanetworkopen.2025.42281)
Supplement: Supplement 2. — Data Sharing Statement [file jamanetwopen-e2542281-s002.pdf]

Data Sharing Statement

McBain. Use of Generative AI for Mental Health Advice Among US Adolescents and Young Adults. *JAMA Netw Open*. Published online November 7, 2025. doi:10.1001/jamanetworkopen.2025.42281

Data
Data available: No

Additional Information
Explanation for why data not available: The data require a Data Use Agreement.
